# Supplementary material for: Single-cell transcriptional profile of CD34+ hematopoietic progenitor cells from del(5q) myelodysplastic syndromes and impact of lenalidomide
Source: Nat Commun. 2024 Jun 20;15:5272. doi: 10.1038/s41467-024-49529-x (PMC11189937; doi:10.1038/s41467-024-49529-x)
Supplement: Supplementary file 1 — Supplementary information [file 41467_2024_49529_MOESM1_ESM.pdf]

Single-cell profiling of del(5q) MDS unveils its transcriptional landscape and the impact of lenalidomide

SUPPLEMENTARY FIGURE LEGENDS

Supplementary Figure 1. Flow cytometry strategy used for the isolation of $CD34^{+}$ cells. SSC-A: side scatter area; FSC-A: forward scatter area; PerCPCy5.5: Peridinin chlorophyll protein-Cyanine5. 5; APC: Allophycocyanin; CD45: cluster of differentiation 45; CD34: cluster of differentiation 34.

Supplementary Figure 2. Hematopoietic $CD34^{+}$ cells from three independent healthy donors were assayed by scRNAseq. **(A)** An overview of the 35,897 cells that passed quality control and filtering for the subsequent analysis in this study. Uniform Manifold Approximation and Projection (UMAP) represents the 13 clusters from 3 integrated healthy donors. HSC: hematopoietic stem cells; LMPP: lymphoid-primed multipotent progenitors; GMP: granulocyte-monocyte progenitors; CLP: common lymphoid progenitor; megakaryocyte-erythroid progenitor; MK_Prog: megakaryocyte progenitor. **(B)** Dotplot showing the percentage and value of the normalized expression of the canonical marker genes used to assign the cell identity to each cluster. **(C)** Independent UMAP for each donor sample colored by cell type. The annotation performed on the integrated assay is transferable to the unique manifold of each sample. **(D)** Barplot representing the contribution of cells from each donor to the different clusters where all the donors, in different proportions, contribute to all clusters except for the pro-B population which has its origin in the healthy donor 3. **(E)** Barplot representing the number of cells assigned to each cell type and the donor of origin for the different cells.

Supplementary Figure 3. Differential expression (DE) analysis between cells labeled as del(5q) and non-del(5q). **(A)** Volcano plot of statistical significance (−log10 Bonferroni-adjusted *p*-values) against fold-change (log2 Fold-change) of gene expression between del(5q) and non-del(5q) cells in all patients. Red points represent genes with $|FC| > 0$ and adjusted p-value < 0.05, and blue points depict genes with $|FC| <0$ and adjusted p-value < 0.05. *p*-values of differentially expressed genes were calculated using the two-sided Wilcoxon signed-rank test. The displayed names of the genes correspond to up to 50 genes located in the CDR. To calculate the enrichment *p*-value, a one-sided hypergeometric test was performed, resulting in a p-value $<2.2e-16$. **(B)** Same

Volcano plots for each patient separately, applying the two-sided Wilcoxon signed-rank test. **(C)** Volcano plots representing the differentially expressed genes within the CDR when cells are arbitrarily shuffled in different percentages (30%, 60%, 80%, 100%). The increase of arbitrarily shuffled cells hampers the detection of differentially expressed genes. Ultimately, when all cell labels are shuffled randomly, the analysis yields no differentially expressed genes.

Supplementary Figure 4. Number of upregulated and downregulated differentially expressed genes obtained in each hematopoietic progenitor for different contrasts. Barplots representing the upregulated (red) and downregulated (blue) genes obtained when comparing: **(A)** del(5q) and **(B)** non-del(5q) cells with healthy donor cells, where del(5q) and non-del(5q) cells came from n=4 biologically independent samples and healthy cells from n=3 biologically independent samples; **(C)** non-del(5q) cells from the complete responder (Patient_6, n=1) and **(D)** the partial responder (Patient_5, n=1) with non-del(5q) cells from patients at diagnosis (n=4); **(E)** non-del(5q) cells from the complete responder (Patient_6, n=1) and **(F)** the partial responder (Patient_5, n=1) with healthy donor cells (n=3); del(5q) cells from the partial responder (Patient_5, n=1) with **(G)** del(5q) cells from patients at diagnosis (n=4) and **(H)** del(5q) cells from the non-responder (Patient_7, n=1); **(I)** del(5q) cells from the non-responder (Patient_7, n=1) with del(5q) cells from patients at diagnosis (n=4). Genes were considered differentially expressed if Benjamini-Hochberg-adjusted p-value < 0.05 and |logFC >2|, except for **(H)**, where they were considered if Benjamini-Hochberg-adjusted p-value < 0.05 and |logFC >0|. HSC: hematopoietic stem cells; LMPP: lymphoid-primed multipotent progenitors; GMP: granulocyte-monocyte progenitors; CLP: common lymphoid progenitor; megakaryocyte-erythroid progenitor; MK_Prog: megakaryocyte progenitor.

Supplementary Figure 5. Gene regulatory network comparative analysis. **(A)** Heatmap showing the dissimilarity score of the regulons calculated for del(5q), non-del(5q) and healthy cells. Warmer color indicates a more different behavior of the regulon between the three phenotypes. Del(5q) and non-del(5q) cells were derived from n=4 del(5q) MDS patients, whereas healthy cells were derived from n=3 healthy donors. Biologically independent replicates (cells) for each phenotype were: del(5q) cells: n=27,117; non-del(5q) cells: n=35,897; healthy cells: n=17,086. HSC: hematopoietic stem cells; LMPP: lymphoid-primed multipotent progenitors; GMP: granulocyte-monocyte progenitors; CLP: common lymphoid progenitor; MEP: megakaryocyte-erythroid progenitor; MK_Prog: megakaryocyte progenitor. **(B)** Dotplot representing

statistically significant biological processes and pathways (Benjamini-Hochberg-adjusted p-value < 0.05) for the target genes forming the regulons of RERE and KDM2A. The one-sided hypergeometric test was used to calculate p-values. Specific dissimilarity score values, as well as the exact p-values for statistically significant biological processes are specified in the Source Data.

Supplementary Figure 6. Functional analysis of the differentially expressed genes before and after treatment. **(A)** Dotplot representing statistically significant biological processes and pathways for differentially expressed genes in del(5q) cells of the partial responder (n=1) compared to del(5q) cells of MDS patients at diagnosis (n=4), and **(B)** for differentially expressed genes in del(5q) cells of the non-responder (n=1) and MDS patients at diagnosis (n=4). Differentially expressed genes were considered as those with Benjamini-Hochberg-adjusted p-value < 0.05 and avgLogFC>2. Enrichment p-values were calculated using a one-sided hypergeometric test from differentially expressed genes determined by two-sided edgeR's Likelihood Ratio Test. Specific p-values for statistically significant biological processes can be found in the Source Data.

Supplementary Figure 1

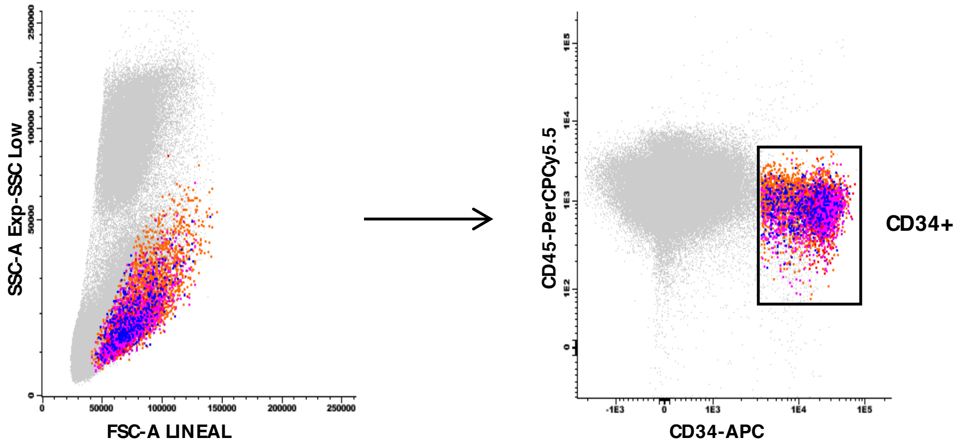

Supplementary Figure 2

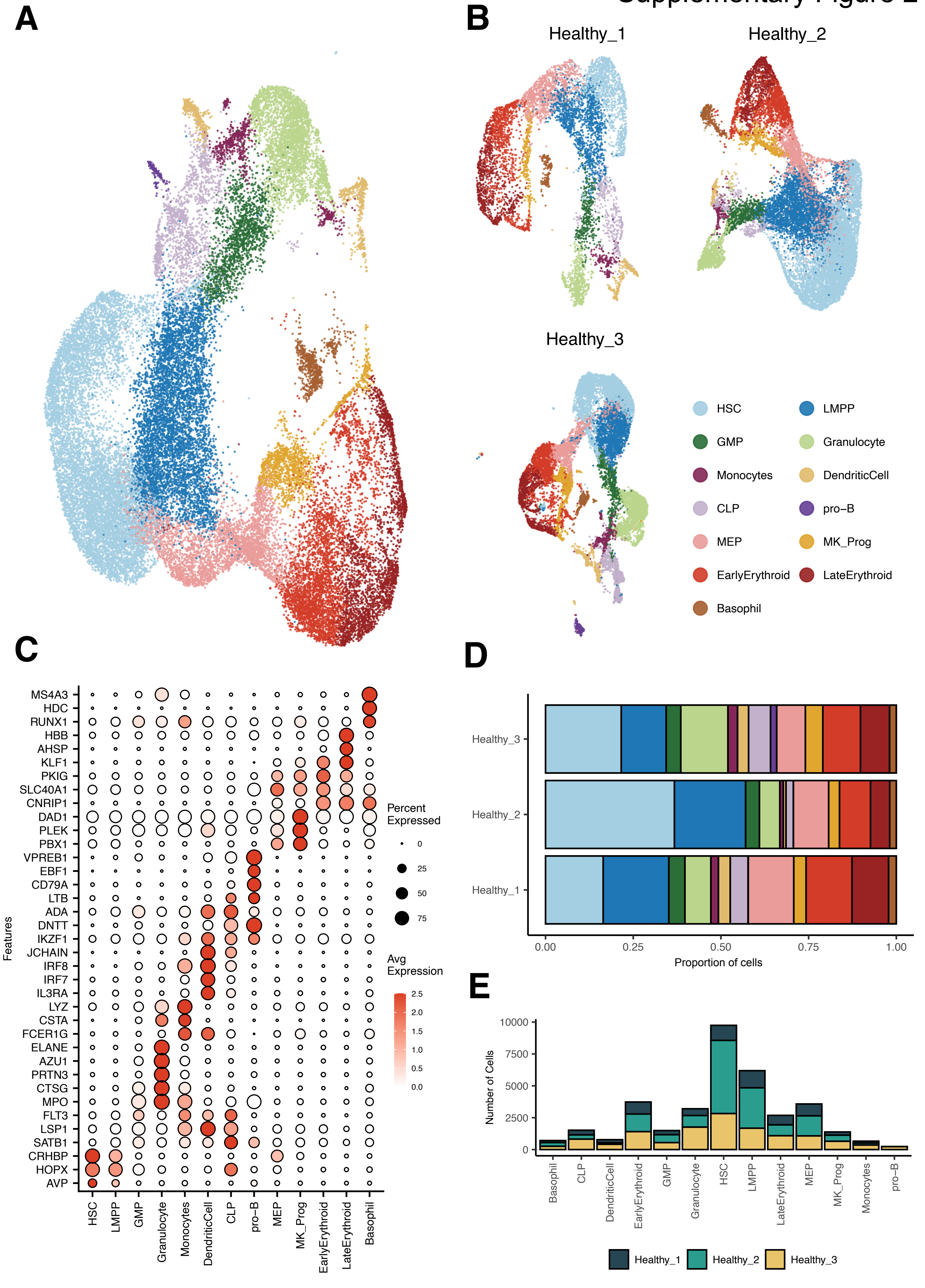

Supplementary Figure 3

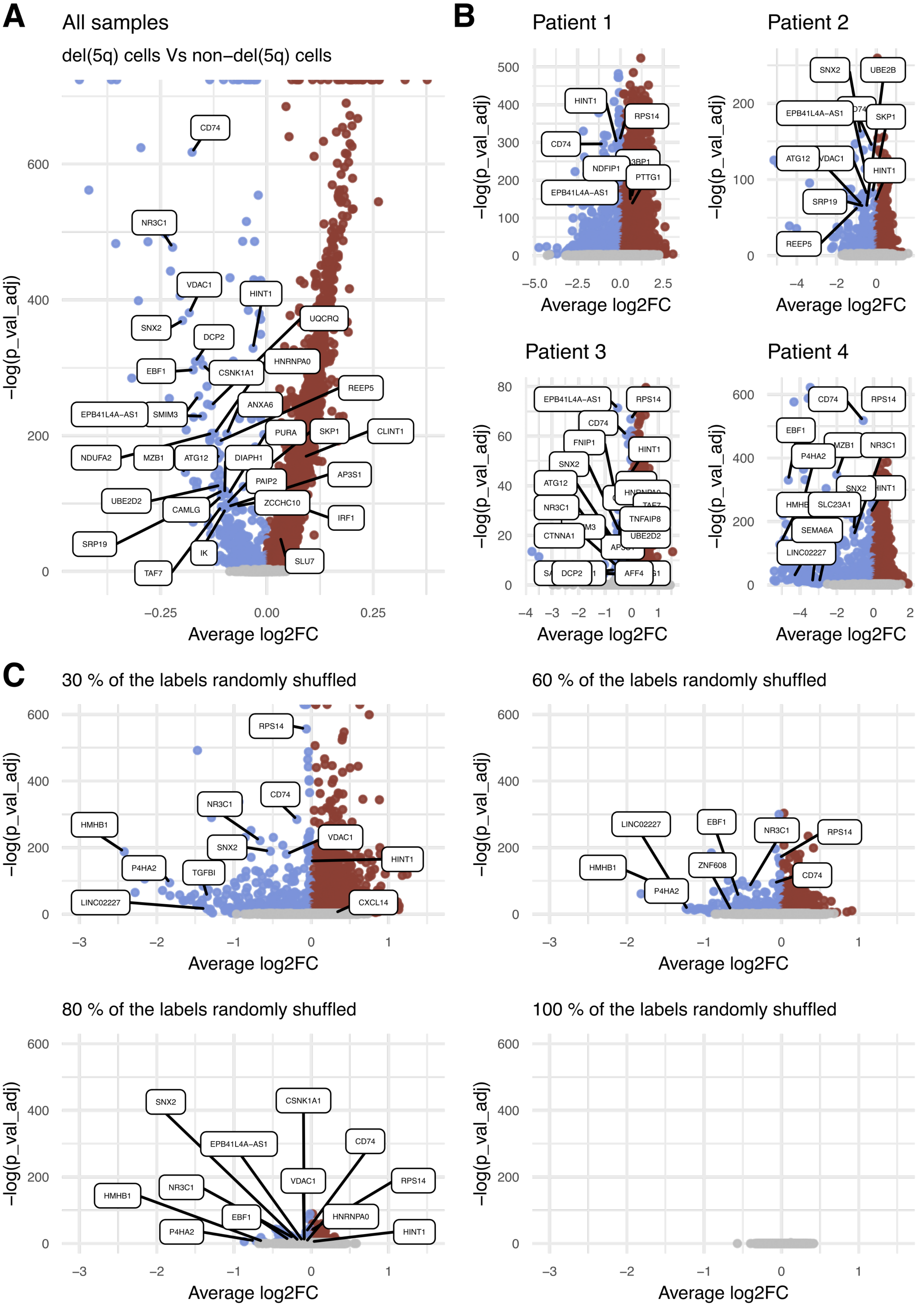

Supplementary Figure 4

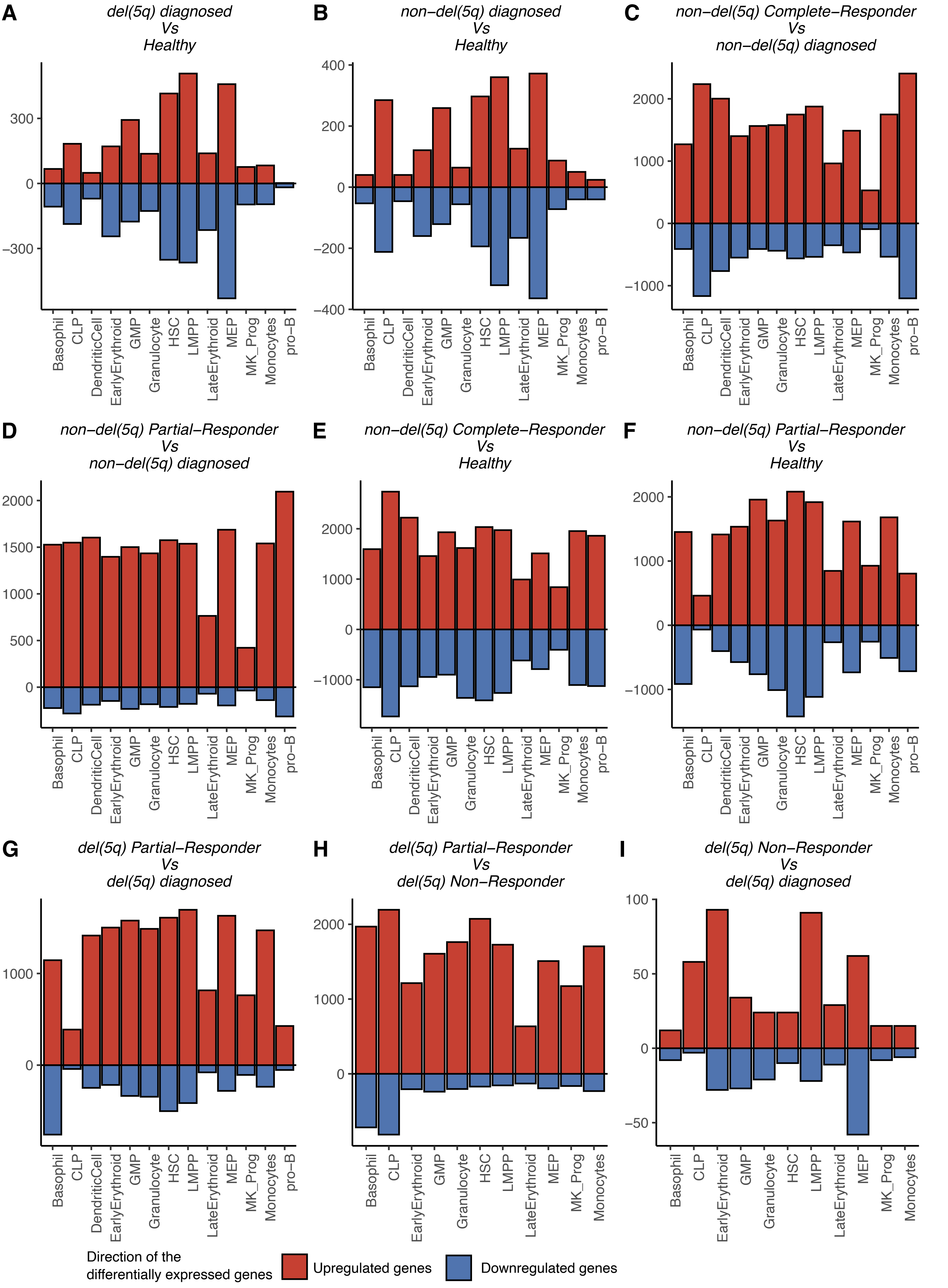

Genes are considered as differentially expressed if: FDR < 0.05 and |logFC| >= 2 except in panel H that are considered if: FDR < 0.05 and |logFC| > 0

Supplementary Figure 5

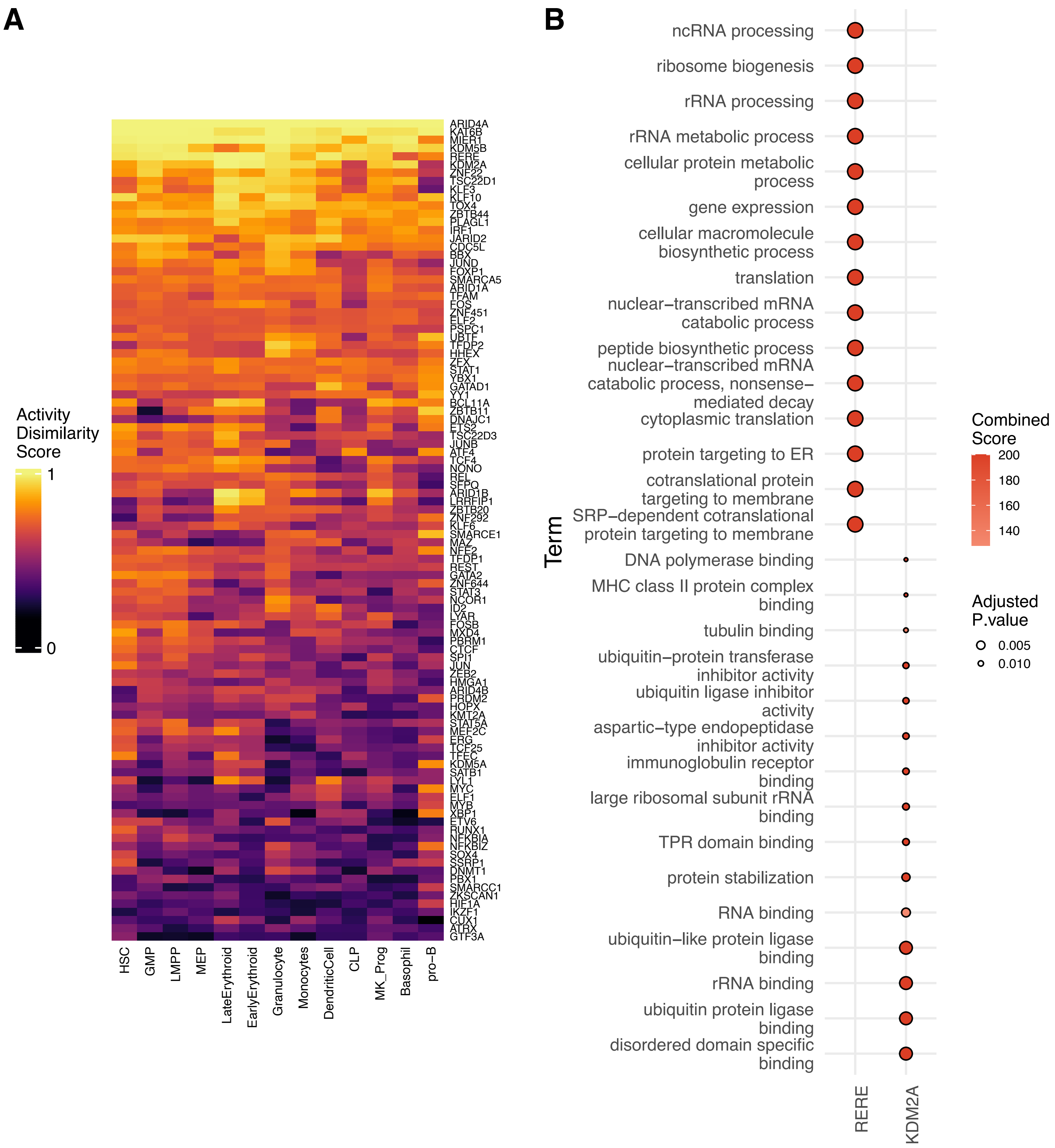

Supplementary Figure 6

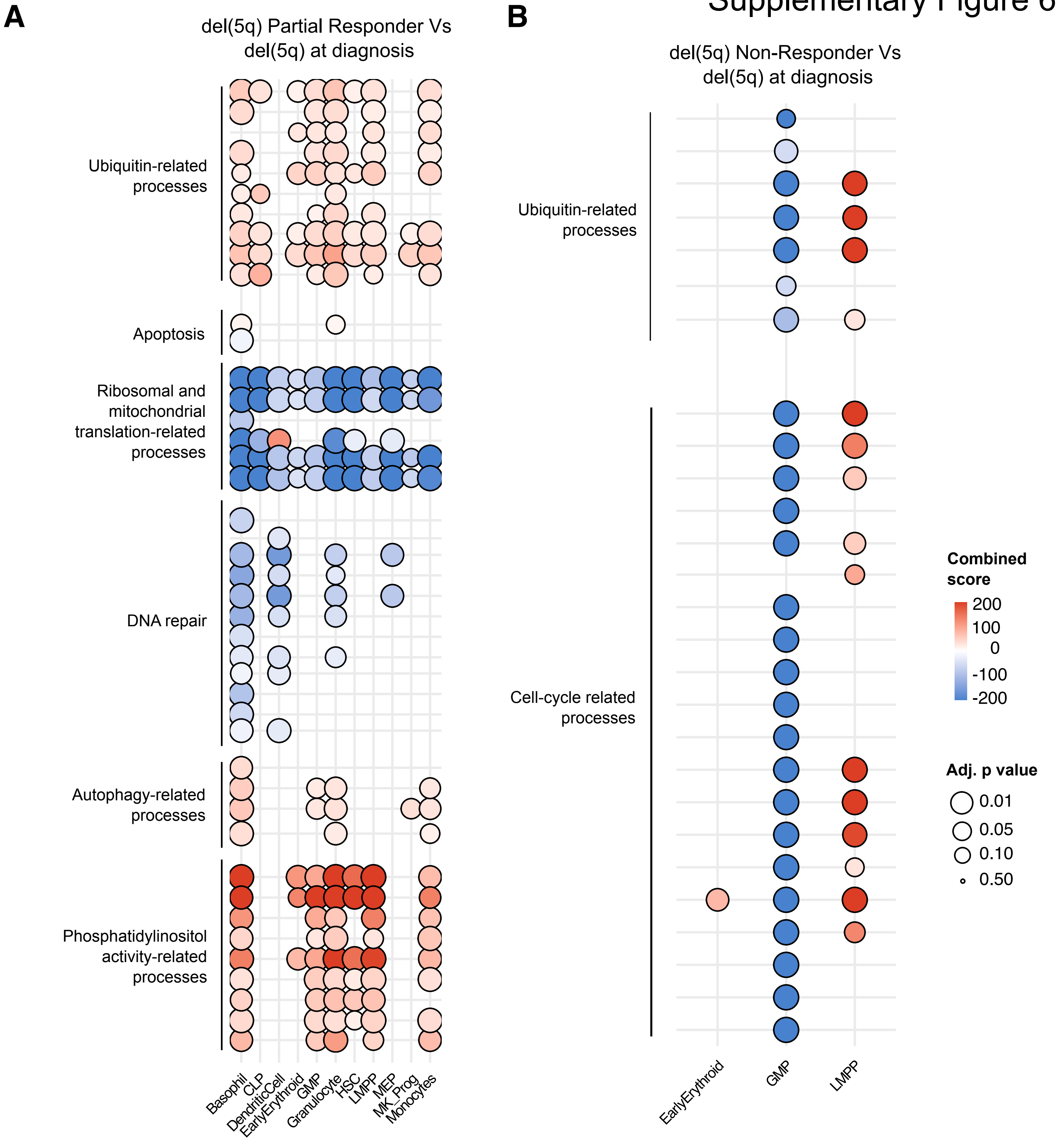

Supplementary Table 1. Clinical information of del(5q) MDS patients and healthy donors.

MDS at diagnosis

Sample ID	Sample Name	Sex	Age (years)	Treatment	Karyotype	Mutations
SMD34459	Patient_1	F	80	Non-treated	46,XX,del(5)(q13q33)[6]/46,XX[8]	*ASXL1* (20%), *SF3B1* (4,6%)
SMD35109	Patient_2	F	91	Non-treated	46,XX,del(5)(q13q33)[15]/46,XX[5]	*TET2* (41%), *DNMT3A* (37%)
SMD35303	Patient_3	F	84	Non-treated	46,XX,del(5)(q13q33)[18]/46,XX[2]	*TET2* (35,6%)
SMD37209	Patient_4	F	81	Non-treated	46,XX,del(5)(q13q35) [7]/46,XX [13]	*SF3B1* (3,91%)

Healthy donors

Sample ID	Sample Name	Sex	Age (years)
GSM5460411	Healthy_1	M	61
GSM5460412	Healthy_2	M	74
GSM5460413	Healthy_3	M	72

MDS treated wit lenalidomide

Sample ID	Treatment response	Sample Name	Sex	Age (years)	Treatment	Karyotype before lenalidomide treatment	Mutations before lenalidomide treatment	Karyotype after lenalidomide treatment	Mutations after lenalidomide treatment
FS-0406	Partial responder	Patient_5	F	75	Lenalidomide +8 cycles	46,XX del(5)(q15q33)[20]	*DNAH9* (35,71%)	46,XX,del(5)(q15q33)[10]/46,XX[10]	*TP53* (7,84%), *DNMT3A* (28,6%)
FS-0634	Complete responder	Patient_6	F	86	Lenalidomide +28 cycles	46,XX,del(5)(q13q33)[15]/46,XX[5]	*CDH13* (47,25%), *DNMT3A* (30,31%)	46,XX [20]	No clinically relevant variants found
SMD132114579	Non-responder	Patient_7	F	87	Lenalidomide +3 cycles	46,XX,del(5)(q21q33)[14]/46,XX[6]	*TP53* (37,8%)	46,XX,del(5)(q21q33)[20]	*DNMT3A* (49,2%), *PPM1D* (48,8%), *TP53* (48,3%)

Supplementary Table 2. Comprehensive breakdown of GO term categorizations from Fig. 7.

**Related to Ubiquitin processes**	Ubiquitin mediated proteolysis<br>Ubiquitin-protein transferase activity (GO:0004842)<br>Ubiquitin-like protein conjugating enzyme binding (GO:0044390)<br>Ubiquitin conjugating enzyme binding (GO:0031624)
**Proteasome mediated processes**	Proteasome-mediated ubiquitin-dependent protein catabolic process (GO:0043161)<br>Proteasomal protein catabolic process (GO:0010498)
**Related to autophagy**	Autophagy<br>Autophagosome assembly (GO:0000045)<br>Autophagosome organization (GO:1905037)
**Erythropoietin signaling**	Signaling By Erythropoietin R-HSA-9006335<br>Erythropoietin Activates Phosphoinositide-3-kinase (PI3K) R-HSA-9027276
**PD-L1/PD-1 pathway in cancer**	PD-L1 expression and PD-1 checkpoint pathway in cancer
**Phosphatidylinositol signaling system**	Phosphatidylinositol signaling system
**Related to ribosomal and mitochondrial translation**	rRNA processing<br>Translation (GO:0006412)<br>Peptide biosynthetic process (GO:0043043)<br>Translational elongation (GO:0006414)<br>Translational termination (GO:0006415)<br>Mitochondrial translation (GO:0032543)<br>Mitochondrial translational termination (GO:0070126)<br>Mitochondrial translational elongation (GO:0070125)

Supplementary Table 3. Comprehensive breakdown of GO term categorizations for PR vs diagnosis contrast from supplementary figure 4.

**Ubiquitin-related processes**	ubiquitin-protein transferase activity (GO:0004842)<br>ubiquitin-like protein ligase activity (GO:0061659)<br>ubiquitin protein ligase activity (GO:0061630)<br>ubiquitin conjugating enzyme activity (GO:0061631)<br>protein polyubiquitination (GO:0000209)<br>protein ubiquitination (GO:0016567)<br>protein K48-linked ubiquitination (GO:0070936)<br>ubiquitin conjugating enzyme binding (GO:0031624)<br>ubiquitin binding (GO:0043130)<br>ubiquitin-like protein conjugating enzyme binding (GO:0044390)
**Apoptosis**	positive regulation of apoptotic process (GO:0043065)<br>regulation of apoptotic process (GO:0042981)
**Ribosomal and mitochondrial translation-related processes**	translation (GO:0006412)<br>translation initiation factor activity (GO:0003743)<br>translational termination (GO:0006415)<br>mitochondrial translational termination (GO:0070126)<br>translational elongation (GO:0006414)<br>mitochondrial translation (GO:0032543)
**DNA repair**	nucleotide-excision repair, DNA incision, 5'-to lesion (GO:0006296)<br>nucleotide-excision repair, DNA incision (GO:0033683)<br>transcription-coupled nucleotide-excision repair (GO:0006283)<br>nucleotide-excision repair (GO:0006289)<br>base-excision repair, gap-filling (GO:0006287)<br>nucleotide-excision repair, DNA incision, 3'-to lesion (GO:0006295)<br>nucleotide-excision repair, preincision complex stabilization (GO:0006293)<br>base-excision repair (GO:0006284)<br>DNA repair (GO:0006281)<br>double-strand break repair (GO:0006302)<br>recombinational repair (GO:0000725)
**Autophagy-related prcesses**	autophagosome assembly (GO:0000045)<br>autophagosome organization (GO:1905037)<br>macroautophagy (GO:0016236)<br>regulation of autophagy (GO:0010506)<br>autophagy of mitochondrion (GO:0000422)
**Phosphatidylinositol activity-related processes**	phosphatidylinositol biosynthetic process (GO:0006661)<br>phosphatidylinositol metabolic process (GO:0046488)<br>phosphatidylinositol phosphate biosynthetic process (GO:0046854)<br>phosphatidylinositol dephosphorylation (GO:0046856)<br>phosphatidylinositol-3-phosphatase activity (GO:0004438)<br>phosphatidylinositol monophosphate phosphatase activity (GO:0052744)<br>1-phosphatidylinositol binding (GO:0005545)<br>phosphatidylinositol-3,5-bisphosphate phosphatase activity (GO:0106018)<br>phosphatidylinositol phosphate kinase activity (GO:0016307)

Supplementary Table 4. Comprehensive breakdown of GO term categorizations for NR vs diagnosis contrast from supplementary figure 4.

**Ubiquitin-related processes**	ubiquitin-protein transferase activator activity (GO:0097027)<br>positive regulation of ubiquitin protein ligase activity (GO:1904668)<br>regulation of ubiquitin protein ligase activity (GO:1904666)<br>positive regulation of ubiquitin-protein transferase activity (GO:0051443)<br>proteasome-mediated ubiquitin-dependent protein catabolic process (GO:0043161)<br>ubiquitin-dependent protein catabolic process (GO:0006511)<br>positive regulation of proteasomal ubiquitin-dependent protein catabolic process (GO:0032436)
**Cell-cycle related processes**	microtubule cytoskeleton organization involved in mitosis (GO:1902850)<br>mitotic sister chromatid segregation (GO:0000070)<br>mitotic metaphase plate congression (GO:0007080)<br>regulation of mitotic cell cycle phase transition (GO:1901990)<br>mitotic nuclear division (GO:0140014)<br>mitotic cell cycle phase transition (GO:0044772)<br>regulation of G2/M transition of mitotic cell cycle (GO:0010389)<br>positive regulation of cell cycle process (GO:0090068)<br>regulation of cell cycle process (GO:0010564)<br>regulation of mitotic cell cycle (GO:0007346)<br>establishment of mitotic spindle localization (GO:0040001)<br>attachment of mitotic spindle microtubules to kinetochore (GO:0051315)<br>sister chromatid segregation (GO:0000819)<br>G2/M transition of mitotic cell cycle (GO:0000086)<br>cell cycle G2/M phase transition (GO:0044839)<br>positive regulation of cell division (GO:0051781)<br>positive regulation of mitotic cell cycle phase transition (GO:1901992)<br>positive regulation of G2/M transition of mitotic cell cycle (GO:0010971)<br>positive regulation of cell cycle G2/M phase transition (GO:1902751)<br>positive regulation of mitotic nuclear division (GO:0045840)
