# Supplementary material for: Single-cell transcriptional profile of CD34+ hematopoietic progenitor cells from del(5q) myelodysplastic syndromes and impact of lenalidomide
Source: Nat Commun. 2024 Jun 20;15:5272. doi: 10.1038/s41467-024-49529-x (PMC11189937; doi:10.1038/s41467-024-49529-x)
Supplement: Supplementary file 5 — Reporting Summary [file 41467_2024_49529_MOESM5_ESM.pdf]

nature portfolio

Corresponding author(s): Teresa Ezponda
Felipe Prosper
Mikel Hernaez

Last updated by author(s): Apr 24, 2024

Reporting Summary

Nature Portfolio wishes to improve the reproducibility of the work that we publish. This form provides structure for consistency and transparency in reporting. For further information on Nature Portfolio policies, see our Editorial Policies and the Editorial Policy Checklist.

Statistics

For all statistical analyses, confirm that the following items are present in the figure legend, table legend, main text, or Methods section.

n/a	Confirmed	
☐	☒	The exact sample size (*n*) for each experimental group/condition, given as a discrete number and unit of measurement
☐	☒	A statement on whether measurements were taken from distinct samples or whether the same sample was measured repeatedly
☐	☒	The statistical test(s) used AND whether they are one- or two-sided<br>*Only common tests should be described solely by name; describe more complex techniques in the Methods section.*
☐	☒	A description of all covariates tested
☐	☒	A description of any assumptions or corrections, such as tests of normality and adjustment for multiple comparisons
☐	☒	A full description of the statistical parameters including central tendency (e.g. means) or other basic estimates (e.g. regression coefficient) AND variation (e.g. standard deviation) or associated estimates of uncertainty (e.g. confidence intervals)
☐	☒	For null hypothesis testing, the test statistic (e.g. *F*, *t*, *r*) with confidence intervals, effect sizes, degrees of freedom and *P* value noted<br>*Give P values as exact values whenever suitable.*
☒	☐	For Bayesian analysis, information on the choice of priors and Markov chain Monte Carlo settings
☒	☐	For hierarchical and complex designs, identification of the appropriate level for tests and full reporting of outcomes
☒	☐	Estimates of effect sizes (e.g. Cohen's *d*, Pearson's *r*), indicating how they were calculated

Our web collection on statistics for biologists contains articles on many of the points above.

Software and code

Policy information about availability of computer code

Data collection	No software was used for data collection
Data analysis	We used the following publicly available software: CopyKat (v1.0.8), CaSpER (v0.2.0), SimiC (v1.0.0), Libra (v1.0.0), edgeR-LRT (v4.0.6), MAST (v.1.22.0), enrichR (v3.2), Liana (v0.1.7), Cell Ranger (v6.0.1), Seurat (v4.2.0), scrublets (v0.2.3), R (v4.2.2), python (v3.6.9).<br>For flow citometry analyses we used BD FACSDIVA v8 software. The code used in this study The code and scripts used in this study are available at https://github.com/GuiSeSanz/MDS_5q_2023/; 10.5281/ZENODO.10983466.

For manuscripts utilizing custom algorithms or software that are central to the research but not yet described in published literature, software must be made available to editors and reviewers. We strongly encourage code deposition in a community repository (e.g. GitHub). See the Nature Portfolio guidelines for submitting code & software for further information.

Data

Policy information about availability of data

All manuscripts must include a data availability statement. This statement should provide the following information, where applicable:

- Accession codes, unique identifiers, or web links for publicly available datasets
- A description of any restrictions on data availability
- For clinical datasets or third party data, please ensure that the statement adheres to our policy

The scRNA-seq data generated in this study have been deposited in the Gene Expression Omnibus database (GEO) under the accession code GSE245452. The

previous publicly available data used in this study, corresponding to scRNA-seq data of CD34+ cells from healthy elderly individuals, are available in GEO under the accession code GSE183328. The GRCh38 assembly of the human genome used is available at NCBI, under the accession code NCBI:GCA_000001405.27. CellPhoneDB database is stored in https://github.com/ventolab/CellphoneDB-data. The biological process and molecular function gene set libraries used for gene ontology analyses are available in https://maayanlab.cloud/Enrichr/enrich. Source data are provided as a Source Data file.

Research involving human participants, their data, or biological material

Policy information about studies with human participants or human data. See also policy information about sex, gender (identity/presentation), and sexual orientation and race, ethnicity and racism.

Reporting on sex and gender	The sex of the healthy donors and MDS patients included in this study is included in supplemental table 1. As del(5q) MDS is a subtype of MDS which is predominantly found in females, and these samples are not very abundant, in the present study we included all the patients for which we were able to obtain samples. Thus, we were not able to select patients by sex or to carry out a sex or gender analysis
Reporting on race, ethnicity, or other socially relevant groupings	No socially relevant categorization values were used in this study.
Population characteristics	The cohort of study included CD34+ cells from healthy elderly controls [(n=3), median age, 72 years, range, 61-84 years] and from patients with MDS [(n=7), median age, 84 years, range, 80-91 years]. Samples from 4 MDS patients were obtained at diagnosis, whereas other 3 were obtained upon lenalidomide treatment: two of them had achieved hematological response (one with partial cytogenetic response, and the other one with complete cytogenetic response), while the third one was a non-responder.
Recruitment	Patients were recruited by their hematologist at the time of diagnosis or upon follow up visits after lenalidomide treatment.
Ethics oversight	Patients provided informed consent, and the study was approved by the Clinical Research Ethics Committee of the Clinica Universidad de Navarra. Patient's data were fully anonymized, and all patients provided informed written consent to have data from their medical records such as age, sex and diagnosis to be used for research purpose.

Note that full information on the approval of the study protocol must also be provided in the manuscript.

Field-specific reporting

Please select the one below that is the best fit for your research. If you are not sure, read the appropriate sections before making your selection.

[x] Life sciences [ ] Behavioural & social sciences [ ] Ecological, evolutionary & environmental sciences

For a reference copy of the document with all sections, see nature.com/documents/nr-reporting-summary-flat.pdf

Life sciences study design

All studies must disclose on these points even when the disclosure is negative.

Sample size	The transcriptome of CD34+ cells was evaluated by single-cell RNA-seq profliing in, healthy elderly donors (n=3), del(5q) MDS patients at diagnosis (n=4), and del(5q) MDS patients upon lenalidomide treatment (n=3). Sample size was determined by specimen availability, and not by statistical predetermination. Nevertheless, to reduce the heterogeneity associated with MDS patients, MDS cohort included only del(5q) patients, excluding cases with ring sideroblasts, excess of blasts, MDS-MLD and MDS-SLD. Sample size used was sufficient to identify del(5q) and non-del(5q) cells in del(5q) MDS patients. Moreover, it allowed the characterization of the transcriptional impact of del(5q) deletion and to define the transcriptional profile non-del(5q) cells in comparison with healthy elderly cells. Additionally, having samples from a complete responder, a partial responder and a non-responder patient to lenalidome allowed us to define the transcriptional effect of lenalidomide both in del(5q) and non-del(5q) cells.
Data exclusions	No data were excluded in this study.
Replication	For differential expression analysis at diagnosis, four del(5q) samples and three samples from elderly individuals were used, each sourced from distinct patient and donor samples. Regarding the lenalidomide-treated samples, we analyzed three separate samples from two hematological responders (one with a partial and one with a complete cytogenetic response) and one non-responder. Due to the varying clinical responses, these samples were studied independently. It is interesting to note that In the case of the hematological responders, similar biological processes were observed, irrespective of the cytogenetic outcome..
Randomization	Not relevant. This study did not involve experimental grouping.
Blinding	Not relevant. This study did not involve experimental grouping.

Reporting for specific materials, systems and methods

We require information from authors about some types of materials, experimental systems and methods used in many studies. Here, indicate whether each material, system or method listed is relevant to your study. If you are not sure if a list item applies to your research, read the appropriate section before selecting a response.

Materials & experimental systems

n/a	Involved in the study
☐	☒ Antibodies
☒	☐ Eukaryotic cell lines
☒	☐ Palaeontology and archaeology
☒	☐ Animals and other organisms
☒	☐ Clinical data
☒	☐ Dual use research of concern
☒	☐ Plants

Methods

n/a	Involved in the study
☒	☐ ChIP-seq
☐	☒ Flow cytometry
☒	☐ MRI-based neuroimaging

Antibodies

Antibodies used: CD34-APC (clone 581; Beckman Coulter #IM2472, lot number 200504, dilution 5:100) and CD45-PerCPCy5.5 (clone HI30; Biolegend #304028, lot number B372674, dilution 1:100),

Validation: All antibodies used have been validated for their use in flow cytometry of human cells.

Plants

Seed stocks: *Report on the source of all seed stocks or other plant material used. If applicable, state the seed stock centre and catalogue number. If plant specimens were collected from the field, describe the collection location, date and sampling procedures.*

Novel plant genotypes: *Describe the methods by which all novel plant genotypes were produced. This includes those generated by transgenic approaches, gene editing, chemical/radiation-based mutagenesis and hybridization. For transgenic lines, describe the transformation method, the number of independent lines analyzed and the generation upon which experiments were performed. For gene-edited lines, describe the editor used, the endogenous sequence targeted for editing, the targeting guide RNA sequence (if applicable) and how the editor was applied.*

Authentication: *Describe any authentication procedures for each seed stock used or novel genotype generated. Describe any experiments used to assess the effect of a mutation and, where applicable, how potential secondary effects (e.g. second site T-DNA insertions, mosiacism, off-target gene editing) were examined.*

Flow Cytometry

Plots

Confirm that:

☒ The axis labels state the marker and fluorochrome used (e.g. CD4-FITC).

☒ The axis scales are clearly visible. Include numbers along axes only for bottom left plot of group (a 'group' is an analysis of identical markers).

☒ All plots are contour plots with outliers or pseudocolor plots.

☐ A numerical value for number of cells or percentage (with statistics) is provided.

Methodology

Sample preparation: For isolation of CD34+ cells from human from bone marrow specimens a red blood cells lysis was performed and cells were stained by incubation with the indicated antibodies for 15 minutes and CD34+, CD45+ progenitor cells were sorted

Instrument: BD FACSAriaTM IIu sorter

Software: Samples were analyzed using the BD FACSDiva software

Cell population abundance: The purity was determined by re-analyzing part of the sorted population by flow cytometry, obtaining >98% CD34+ cells.

Gating strategy: For isolation of CD34+ cells, cells were gated by size (FSC/SSC), and sorted based in CD34 and CD45 expression (CD34 positive expression and CD45 medium expression).

☒ Tick this box to confirm that a figure exemplifying the gating strategy is provided in the Supplementary Information.
